# RBFOX and PTBP1 proteins regulate the alternative splicing of micro-exons in human brain transcripts

**DOI:** 10.1101/gr.181990.114

**Published:** 2015-01

**Authors:** Yang I. Li, Luis Sanchez-Pulido, Wilfried Haerty, Chris P. Ponting

**Affiliations:** 1 MRC Functional Genomics Unit, Department of Physiology, Anatomy and Genetics, University of Oxford, Oxford OX1 3PT, United Kingdom;; 2 Wellcome Trust Centre for Human Genetics, University of Oxford, Oxford OX3 7BN, United Kingdom

## Abstract

Ninety-four percent of mammalian protein-coding exons exceed 51 nucleotides (nt) in length. The paucity of micro-exons (≤ 51 nt) suggests that their recognition and correct processing by the splicing machinery present greater challenges than for longer exons. Yet, because thousands of human genes harbor processed micro-exons, specialized mechanisms may be in place to promote their splicing. Here, we survey deep genomic data sets to define 13,085 micro-exons and to study their splicing mechanisms and molecular functions. More than 60% of annotated human micro-exons exhibit a high level of sequence conservation, an indicator of functionality. While most human micro-exons require splicing-enhancing genomic features to be processed, the splicing of hundreds of micro-exons is enhanced by the adjacent binding of splice factors in the introns of pre-messenger RNAs. Notably, splicing of a significant number of micro-exons was found to be facilitated by the binding of RBFOX proteins, which promote their inclusion in the brain, muscle, and heart. Our analyses suggest that accurate regulation of micro-exon inclusion by RBFOX proteins and PTBP1 plays an important role in the maintenance of tissue-specific protein–protein interactions.

Most vertebrate pre-mRNA are divided into short exonic sequences separated by longer intronic stretches that are removed during mRNA maturation. Although alternative splicing of exons allows multiple protein isoforms to be produced from the same gene, many isoforms appear to lack functional roles, owing to their low evolutionary conservation and expression levels (Melamud and Moult 2009; Pickrell et al. 2010; Reyes et al. 2013). Consequently, the distinction of functional isoforms from those that serve no protein-encoded function represents considerable challenges not just for genomics researchers but perhaps also for the cellular splicing machinery.

Exons exhibit widely diverse characteristics and functions. They differ greatly in their nucleotide composition (Amit et al. 2012), inclusion pattern (Keren et al. 2010), and length (Sorek et al. 2004), all of which can affect their biological roles and how they are recognized during splicing. The length of an exon is often assumed to follow a symmetric distribution centered around an optimal size, which is ∼140 nucleotides (nt) in mammals (Berget 1995; Zhu et al. 2009; Gelfman et al. 2012). This size is proposed to relate to the amount of DNA wrapped around single nucleosomes (Schwartz et al. 2009). The preferential positioning of nucleosomes within exons is hypothesized to aid exon recognition by slowing down RNA polymerase II, thereby allowing more time for the splicing machinery to assemble and splice out the intron immediately upstream (Schwartz et al. 2009).

In contrast to ordinary exons, ultra-short exons are relatively uncommon and often have unknown functional roles. Several observations lend support to the hypothesis that micro-exons (exons of length ≤ 51 bp) are difficult for the splicing machinery to process and, consequently, that there is significant selective pressure on exon lengths to remain longer than 51 nt. Here, we study exons 51 nt or shorter because of their increased tendency to be skipped in mature transcripts, possibly because molecular crowding between large multimeric complexes cause steric hindrance that inhibits spliceosome assembly (Black 1991; Dominski and Kole 1991; Carlo et al. 1996, 2000; Simpson et al. 2000). Indeed, experimental shortening of a constitutively spliced internal exon in a synthetic gene construct to 51 nt or shorter induced skipping phenotypes (Dominski and Kole 1991). It is also commonly believed that exons must possess a minimum number of exonic splicing enhancers that promote binding of splicing factors in order to be accurately spliced (Blencowe 2000; Fairbrother et al. 2002; Caceres and Hurst 2013), a requirement that is increasingly more difficult to meet as exon size decreases. Lastly, we note that the number of annotated exons declines sharply as their sizes decrease below 100 nt (Fig. 1B).

**Figure 1.**
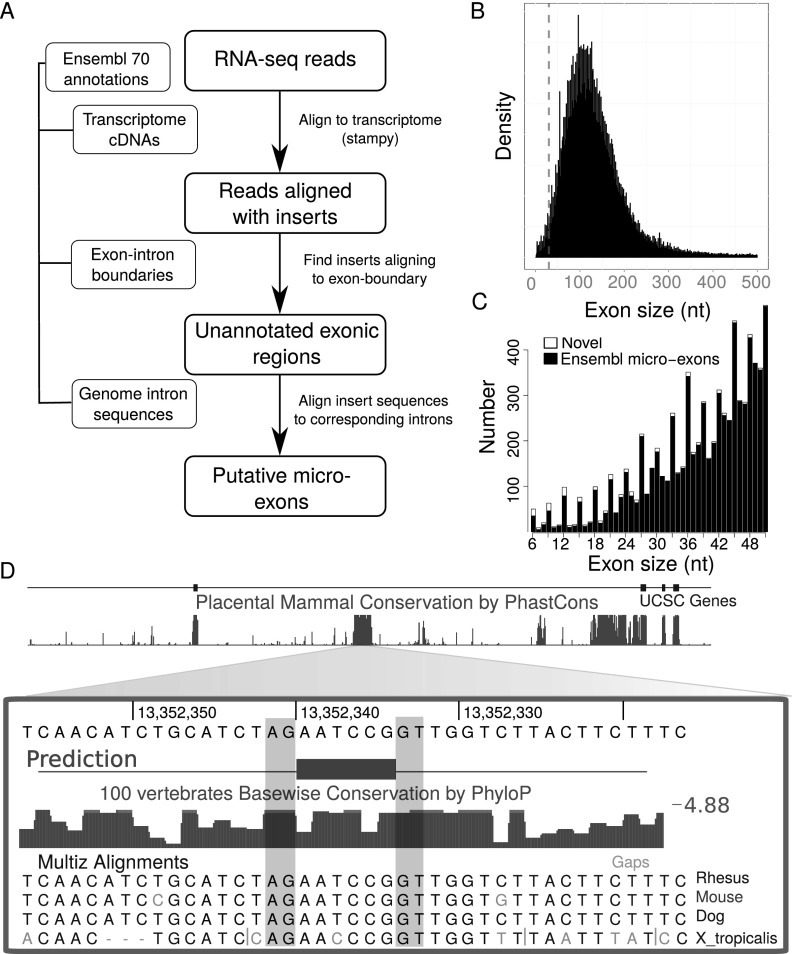
Identification of novel micro-exons. (*A*) Flowchart of our micro-exon discovery pipeline. Ensembl release 70 annotation was first used to build all cDNA transcripts on which RNA-seq reads were mapped using Stampy (Lunter and Goodson 2011). Reads aligning with insertions of up to 51 nt in length were then scanned to identify those whose insertions aligned to exon-exon boundaries. Subsequently, the inserted sequences were aligned to the intronic sequences separating the corresponding exons. Putative novel micro-exons were then defined as exons that were flanked by canonical splice sites and were supported in at least 15% of all samples. (*B*) The density of internal exon sizes shows that the majority is distributed around 140 nt in length, while there is a sharp decrease in the number of exons shorter than 51 nt (dashed line) as exon size decreases. (*C*) Previously annotated micro-exons from Ensembl release 70 that show evidence for expression in brain samples (black) compared to novel predicted micro-exons expressed in brain samples (white). Although the annotation of internal exons of sizes 22–51 nt appears to be nearly complete, we identified a large number of novel micro-exons between 6 and 21 nt in length. (*D*) Example of a novel predicted micro-exon. This micro-exon is only 6 nt in length and lies within a conserved region of the *CACNA1* gene. The splice sites of this micro-exon are conserved in mammals and in *Xenopus*.

Despite these apparent constraints on exon sizes, functional micro-exons have been previously identified (Carlo et al. 2000; Zibetti et al. 2010). For example, the inclusion of a 12-nt micro-exon in *KDM1A* is regulated by PTBP1 (polypyrimidine tract binding protein) in a brain-specific manner (Xue et al. 2013) and contributes to neurite morphogenesis in mammals (Zibetti et al. 2010). The existence of functional micro-exons as short as these raises two major questions: (1) How are very short exons recognized and processed by the cell despite appearing to be disfavored? (2) Are there functional roles specific to micro-exons that are not shared with longer exons? Furthermore, it is as yet unknown whether functional micro-exons are common, or are oddities among more prevalent cases of noisy splicing and annotation errors.

Here, we provide a comprehensive characterization of micro-exons at the DNA conservation, RNA-splicing, and protein tertiary structure levels. We analyzed more than 57 billion reads from 901 human and mouse RNA-seq libraries including 25 postmortem human brain samples across development (Mazin et al. 2013), 345 samples from human postmortem prefrontal cortices (Lonsdale et al. 2013), and 531 samples from diverse human and mouse tissues (Merkin et al. 2012; Lonsdale et al. 2013); Illumina Human BodyMap Project (European Nucleotide Archive [ENA; http://www.ebi.ac.uk/ena/]; accession number ERA022994) to show that thousands of micro-exons are highly conserved across vertebrates and mammals at the sequence and exon inclusion levels, respectively. Analysis of 7949 brain-expressed micro-exons revealed that constitutively spliced (CS) micro-exons possess strong genomic signatures predicted to facilitate splicing, including stronger splice-site motifs, shorter flanking introns, and a higher density of exonic splicing enhancers compared to longer exons. In contrast, alternative spliced micro-exons are flanked by highly conserved intronic flanks that harbor intronic splicing enhancers including RNA motifs bound by RBFOX and PTBP1 proteins. We found that micro-exons regulated by RBFOX proteins are characterized by weaker splice sites, longer flanking introns and lower exonic splicing enhancer densities than other micro-exons. These observations indicate that RBFOX proteins can facilitate the splicing of micro-exons. We also found that PTBP1 likely regulates the inclusion of micro-exons, possibly by repressing the inclusion of micro-exons that are enhanced by RBFOX proteins and other splicing factors. Our analysis is the first, to our knowledge, to provide examples of how alternatively spliced (AS) micro-exons can impact cellular functions either by affecting post-transcriptional regulation or by regulating protein–protein interactions through changes in protein tertiary structure.

## Results

### Discovery of micro-exons and quantification of their usage

A total of 12,835 Ensembl-annotated internal micro-exons were identified in protein-coding genes (Fig. 1C). To complement this Ensembl annotation (Flicek et al. 2014), we identified a further 310 novel putative micro-exons between 6 and 51 nt from a large number of available RNA-seq data sets from human brain (307 samples), muscle (74 samples), and nerve (47 samples) using a discovery pipeline (Fig. 1A; Methods). This indicates that most human micro-exons are already known and that our analysis is representative of all micro-exons. We then sought to assess the validity of our novel predicted micro-exons by analyzing their sequence conservation in comparison to those of Ensembl-annotated micro-exons (Supplemental Fig. 1A). Their high levels of sequence conservation justified the consideration in subsequent analyses of both novel and previously annotated micro-exons, a total of 13,095 micro-exons. We also identified several novel micro-exons whose lengths were shorter than 6 nt. For instance, we identified a 3-nt micro-exon in *TLN1* that is conserved in mammals (Supplemental Fig. 2). However, > 20% of micro-exons shorter than 3 nt mapped exactly to multiple positions within intronic sequences (Methods). Although it may be possible to use intron conservation to predict AS micro-exons that are shorter than 6 nt, they were discarded from further analysis because we expected 6- and 9-nt micro-exons, which we were able to detect, to possess similar splicing mechanisms as 3-nt micro-exons.

Despite the small number of novel (non-Ensembl) micro-exons discovered, several among our set of 310 (Supplemental File 1) lie within genes previously associated to human diseases or other genetic traits. After discarding 30 exons that were previously annotated in three additional databases (GENCODE v19, UCSC, and RefSeq), 64 novel micro-exons were contained in genes linked to at least one disease in the Online Mendelian Inheritance in Man database (OMIM 2014). Among micro-exons alternatively spliced in a disease-associated gene was a 6-nt micro-exon that we could map to a highly conserved region of the *CACNA1A* gene (Fig. 1D). This encodes a calcium channel, voltage-dependent, P/Q type, alpha 1A subunit that is mutated in spinocerebellar ataxia type 6, a familial hemiplegic migraine and episodic ataxia type 2 (MIM 108500, 141500, and 183086). We were able to map the two residues (NP) that are encoded by this AS micro-exon to a loop linking the S3 and S4 regions of CACNA1A (Payandeh et al. 2012). According to Payandeh et al. (2012), this loop has a dynamic connection to S4 and moves during channel gating. The alternative inclusion of the micro-exon may therefore generate two *CACNA1A* isoforms with contrasting gating kinetics.

Nearly half (136 or 43.9%) of our novel micro-exons were 6–21 nt in length compared to only 12.3% (1575) of Ensembl-annotated micro-exons. This prompted us to investigate the ability of algorithms to detect and accurately map RNA-seq reads onto micro-exons. Mapping RNA-seq reads directly onto the genome is computationally difficult owing to a large search space for small exons. We expected reads spanning micro-exons (and thus three or more exons) to further complicate the mapping procedure. We therefore compared the ability of several RNA-seq aligners, including STAR (Dobin et al. 2013), TopHat2 (Kim et al. 2013), and OLego (Wu et al. 2013), to map reads onto micro-exons of decreasing sizes (Methods). Compared to a micro-exon mapping method we developed (ATMap or Augmented Transcriptome Mapping; see Methods), both TopHat2 and STAR aligners mapped fewer reads onto short micro-exons, while all four methods mapped similar numbers of reads to larger exons (Supplemental Fig. 3). In particular, ATMap mapped more reads to micro-exons of sizes 9–21 bp (median log_2_ fold differences of 0.55–4.00) compared to TopHat2 and STAR (Supplemental Fig. 3). OLego’s performance was similar to ATMap’s (median log_2_ fold differences of 0.48–0.92). We therefore used ATMap to quantify the usage of novel and previously annotated micro-exons across all 901 RNA-seq samples (Methods).

### Micro-exons possess tissue-specific inclusion patterns

**Figure 2.**
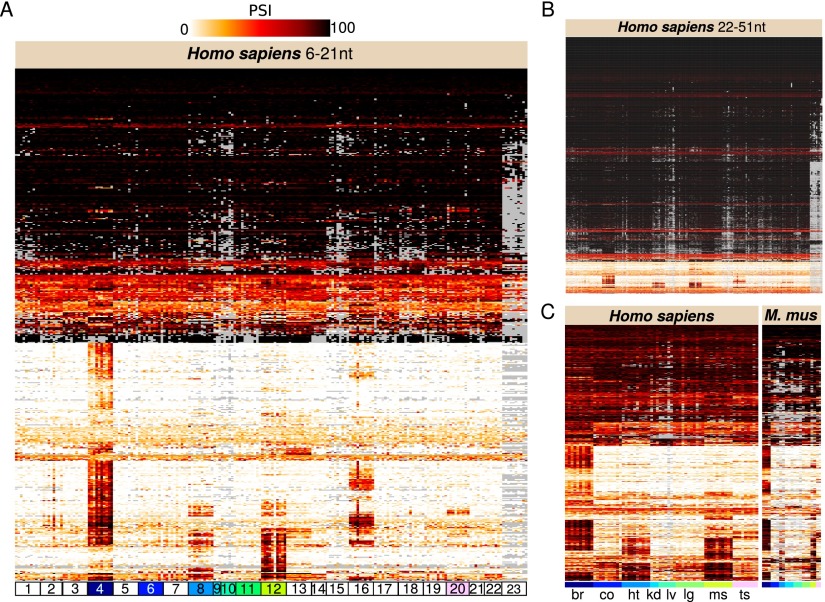
Tissue-dependent inclusion of micro-exons. (*A*) Inclusion rates of 534 micro-exons of length 6–21 nt and (*B*) 5158 micro-exons of length 22–51 nt in 23 tissues. 1: adipose, 2: adrenal, 3: artery, 4: brain, 5: breast, 6: colon, 7: esophagus, 8: heart, 9: kidney, 10: liver, 11: lung, 12: muscle, 13: nerves, 14: ovary, 15: pancreas, 16: pituitary, 17: prostate, 18: skin, 19: stomach, 20: testis, 21: thyroid, 22: uterus, and 23: whole blood. Colored samples are compared to matched mouse samples (*C*). The inclusion rates of 431 human alternatively spliced micro-exons and their mouse orthologs in eight tissues. *Left* to *right*: (br) brain, (co) colon, (ht) heart, (kd) kidney, (lg) lung, (lv) liver, (ms) muscle, and (ts) testis. In all three heatmaps, rows (micro-exons) were clustered according to their PSI profiles. (Gray) Micro-exons for which PSI could not be computed because of insufficient number of reads (≤ 5) spanning splice junctions.

We quantified the splicing inclusion ratios of micro-exons across all RNA-seq samples assigning PSI (percent spliced-in) values for each micro-exon and sample. Given our initial assumption that micro-exons tend to be skipped, we observed a surprisingly large proportion of micro-exons (∼81%) that were constitutively spliced across all tissues (CS; median PSI ≥ 90) (Fig. 2A,B). However, consistent with our expectation, the number of CS micro-exons sharply decreases with exon size, whereas the number of AS micro-exons appears to remain approximately constant as exon size decreases (Fig. 2A; Supplemental Fig. 4).

We also observed that the splicing ratios of AS micro-exons were consistent across human tissues (Fig. 2A,B), which suggests they possess widespread and basic cellular functions. Many AS micro-exons appeared to be used in the brain, heart, muscle, or pituitary gland but not in other tissues (Fig. 2A,B). Noticeably, we identified 145 micro-exons that were included in brain-expressed transcripts, but skipped in nearly all other tissue samples (Methods; Supplemental Fig. 5C), suggesting that they may possess brain-specific functions. For example, a highly conserved 24-nt micro-exon in *CPEB4*, encoding a key RNA-binding protein that controls cytoplasmic polyadenylation (Huang et al. 2006), is included in neural transcripts alone (see Supplemental File 2 for a complete list). In total, 7949 (of 13,085) micro-exons were included in transcripts expressed in the brain. Of these, 6469 (81.4%) micro-exons were CS (brain median PSI ≥ 90) and the remaining 1480 were AS (brain median PSI between 10 and 90) in the brain (Methods). Reassuringly, the inclusion ratios of micro-exons in the brain were replicated in GTEx brain samples, in samples from developing and aging brains (Mazin et al. 2013), and in the Illumina Human BodyMap project brain samples (Supplemental Fig. 5A,B). This observation allows us to exclude potential technical artifacts such as batch effects and protocol-specific biases when explaining the data, and suggests a constant usage of most micro-exons across human brain development and aging (Supplemental Fig. 5A,B).

We next identified evidence that brain-specific patterns of micro-exon usage have been largely preserved across the ∼90 million years that separate human and mouse lineages. For this, we retrieved 23 mouse RNA-seq samples from brain and seven other organs (Merkin et al. 2012) and quantified the inclusion ratios of micro-exons previously annotated within the mouse genome. We then compared these inclusion ratios to those of the orthologous micro-exons in human. Owing to the small number of micro-exons annotated previously in the mouse genome and the shallower depth of mouse RNA-seq data, we were only able to quantify the inclusion ratios of 617 micro-exon orthologs (out of 1581) that are alternatively spliced in human brains (Methods). Nevertheless, we observed a clear correspondence between the micro-exon inclusion patterns of human and mouse brains (Fig. 2C). As expected (Merkin et al. 2012), micro-exon usage is more similar among human brain samples (Pearson correlation: 0.77–0.92; Supplemental Fig. 6), than it is between human and mouse brain samples (Pearson correlation: 0.61–0.76). In contrast, the correlations of micro-exon usage between human brain samples and samples from other human tissues, or those between mouse brain samples and samples from other mouse tissues are significantly lower (0.32–0.67 and 0.35–0.51, respectively).

The general tendency of micro-exons to be expressed in the brain and the high conservation of their inclusion levels between human and mouse brains suggest that micro-exons may be particularly important for brain function.

### Most micro-exons are well conserved in vertebrates

**Figure 3.**
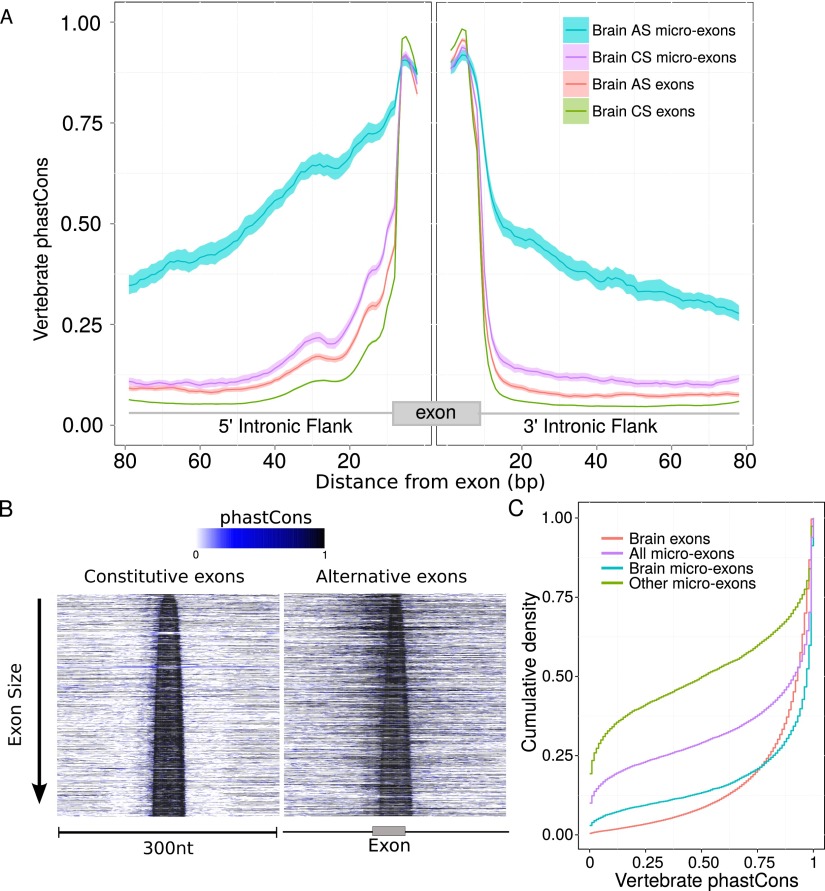
Conservation of micro-exons across vertebrates. (*A*) The intronic conservation is significantly higher for AS micro-exons than for other classes of exons. Brain micro-exon sequences are highly conserved. Mean vertebrate phastCons scores and 95% confidence interval in the intronic flanks of symmetric (multiples of three nucleotides) alternatively spliced (AS) micro-exons, constitutively spliced (CS) micro-exons, all AS exons, and all CS exons. (*B*) A large proportion of AS micro-exons show conservation in their intronic flank in addition to strong conservation within exonic sequences. Vertebrate phastCons scores of 1500 randomly sampled CS and AS micro-exons sorted by size and centered within a 300-nt window. (*C*) Brain expressed micro-exon sequences are highly conserved. Cumulative density of average phastCons score of all exons expressed in the brain (red), all annotated micro-exons (purple), all annotated micro-exons with brain usage (teal), and all annotated micro-exons without evidence of brain usage (green).

Sequence conservation has been widely used as a proxy for functionality (Hardison 2003). We therefore hypothesized that most micro-exons should show evidence for increased sequence conservation relative to neutrally evolving sequences. Indeed, ∼60% of all 13,095 micro-exons assessed had a mean phastCons score of 0.8 or higher. By comparison, ∼8.2% of the bases in the human genome are thought to be under selective constraint (Rands et al. 2014), yet < 5% possess a phastCons score higher than 0.8 (Siepel et al. 2005). This observation therefore suggests that the majority of annotated micro-exons are likely to be functional. Furthermore, the 7949 micro-exons with evidence of inclusion in the brain were far better conserved than the 5146 remaining micro-exons with no or weak evidence of brain usage. About 75% of all brain-expressed micro-exons had an average phastCons score of 0.8 or higher (Fig. 3C). In contrast, ∼43% of Ensembl-annotated micro-exons with weak or no evidence of brain expression had an average phastCons score of 0.2 or lower (< 5% of RefSeq CDS bases score < 0.2) (Siepel et al. (2005). Thousands of Ensembl-annotated micro-exons may therefore be either annotation errors or annotated products of noisy splicing (Pickrell et al. 2010).

These observations motivated us to focus on the 7949 brain-expressed micro-exons. We next identified 97,816 and 10,306 longer exons that were CS and AS in the brain, respectively, in order to compare with our previously defined sets of 6469 and 1480 CS and AS micro-exons, respectively. In terms of conservation, both AS and CS micro-exons were highly conserved across vertebrates and possessed similar levels of conservation as longer exons (Fig. 3A,B; Supplemental Figs. 1B, 7). Intriguingly, we found that intronic flanks of symmetric (exon-phase symmetric) AS micro-exons (i.e., exons of length exactly divisible by three) are highly conserved, while the flanks of nonsymmetric (not exon-phase symmetric) AS micro-exons were conserved at lower levels (Supplemental Fig. 8), in contrast to other exon classes whose flanks showed nearly no vertebrate conservation (phastCons score < 0.2) (Fig. 3A). The elevated conservation flanking AS micro-exons extends to > 75 bp within each intronic flank and may harbor conserved regulatory sequences that enhance their splicing.

### CS micro-exons possess genomic features that enhance splicing

To understand how the 7949 brain-expressed micro-exons can be accurately recognized and spliced within the cell, we asked whether micro-exons require particular genomic features that facilitate recognition and processing by the splicing machinery. Several exonic properties are known to enhance splicing including shorter flanking introns (Sterner et al. 1996; Hertel 2008), stronger splice site motifs (Yeo and Burge 2004), and higher densities of splicing enhancers (Graveley 2000; Wang et al. 2004). The combined effect of these properties likely defines exonic splicing efficiency. To compensate for splicing difficulties arising from their short sizes, we hypothesized that micro-exons possess stronger splicing-enhancing genomic features compared to longer exons.

**Figure 4.**
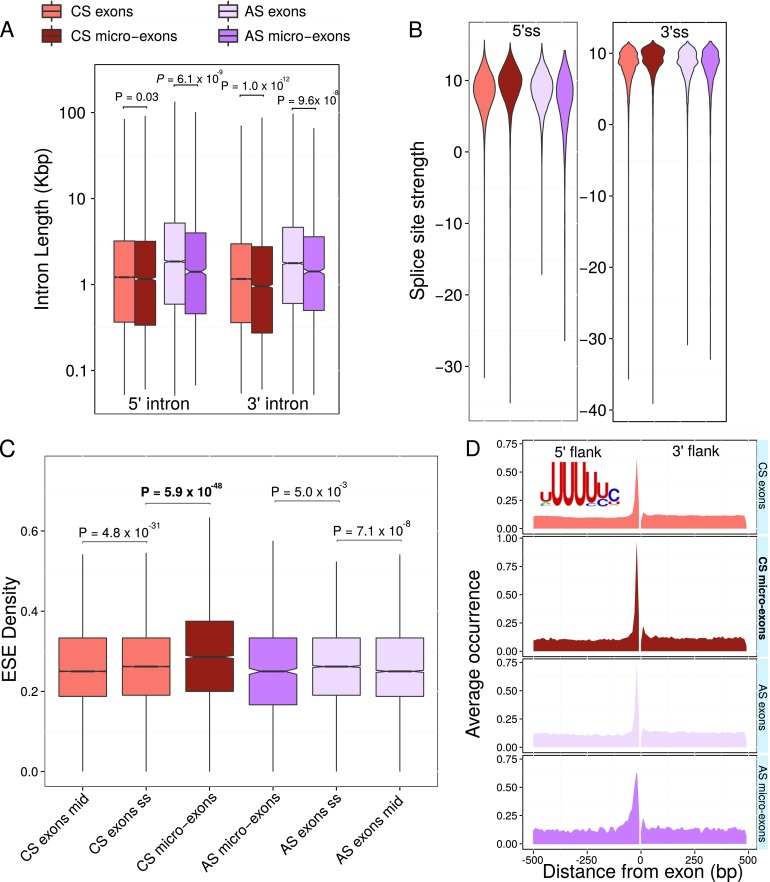
Genomic features of constitutively spliced micro-exons enhance splicing. (*A*) Introns flanking micro-exons are significantly shorter than those of longer exons. Length distribution of intron lengths flanking different classes of exons. (*B*) The 5′ and 3′ splice site motifs of CS micro-exons are significantly stronger than the 5′ and 3′ splice site motifs of other classes of exons, respectively. Strengths of 5′ and 3′ splice sites according to MAXENT scores in alternatively spliced, constitutively spliced micro-exons and in longer alternatively spliced and constitutively spliced exons. (*C*) CS micro-exons, but not AS micro-exons, have significantly higher densities of exonic splicing enhancers (ESE) than longer exons. Comparison of the densities of 607 previously established exonic splicing enhancer hexamers (Fairbrother et al. 2002; Zhang and Chasin 2004; Goren et al. 2006) in the central 24 nt of CS or AS exons longer than 100 bp (CS or AS exons mid), and in 12 nt of exonic sequence adjacent to the 5′ and 3′ splice sites (24 nt in total) of CS or AS exons longer than 100 bp (CS or AS exons ss), and in whole CS and AS micro-exons. (*D*) The binding motif of U2AF2 (*top left*) appears to be highly concentrated in the polypyrimidine tract immediately upstream (10–20 nt upstream) of all classes of exons. This enrichment is highest, however, in CS micro-exons (0.96 motifs by exon vs. 0.61–0.75).

As predicted, we identified several features of micro-exons predicted to facilitate splicing. For example, introns flanking AS and CS micro-exons tend to be shorter than those flanking longer AS and CS exons, respectively, with the most significant difference between the lengths of 3′ introns of CS micro-exons and those of longer CS exons (median 955 nt vs. 1161 nt, *P* *<* 1.02 × 10^−12^ Mann-Whitney *U* test) (Fig. 4A). Furthermore, 5′ and 3′ splice sites for CS micro-exons (but not longer exons or AS micro-exons) were also found to have unusually high signal strength as measured by MaxEntScan (Yeo and Burge 2004) score (5′: median 9.59 vs. 7.83–8.81, *P* *<* 1.1 × 10^−74^; 3′: median 9.22 vs. 8.6–8.73, *P* *<* 1.2 × 10^−60^ Mann-Whitney *U* test) (Fig. 4B). Notably, we found that the density of 607 exonic splicing enhancers determined by previous studies (Fairbrother et al. 2002; Zhang and Chasin 2004; Goren et al. 2006) was significantly higher in CS micro-exons compared to that in longer CS exons, even exceeding their densities in exonic sequence immediately flanking splice sites (median 0.286 vs. 0.262, *P* *<* 5.9 × 10^−48^ Mann-Whitney *U* test, Methods) (Fig. 4C). Differences in length distribution between AS and CS micro-exons could contribute to these observations. Nevertheless, because there was no significant association between exon length and exonic splicing enhancer density for exons smaller than 50 nt (Supplemental Fig. 9A), exonic splicing enhancer densities indeed appear to be different between AS and CS micro-exons. We did, however, observe an increasing trend for MaxEntScan score as exon size decreases (Supplemental Fig. 9B), which is consistent with our hypothesis that CS micro-exons require stronger splice sites than other exons to be efficiently recognized.

We also observed an increased thymine content 5–20 bp upstream of CS micro-exons compared to other classes of exons (Supplemental Fig. 10). When we calculated the median nucleotide content, we found a higher proportion of thymine immediately upstream of CS micro-exons (but not of AS micro-exons) immediately (5–20 bp) upstream of the start site. This increase in thymine content does not extend further into the upstream intron (Supplemental Fig. 10). Consequently, we expect that the polypyrimidine tracts are located downstream from the branch point for CS micro-exons. In contrast, AS micro-exons showed lower proportions of thymine content immediately upstream, but appeared to possess higher thymine contents > 20 nt into the upstream intron, and overall. These observations are consistent with the notion that different splicing mechanisms contribute to CS and AS micro-exon regulation.

Because higher thymine and cytosine content can strengthen polypyrimidine tracts and enhance splicing, we searched for thymine-rich motifs within a compendium of RNA-binding motifs (Ray et al. 2013) that are more strongly enriched upstream of CS micro-exons compared to other classes of exons. As expected, several thymine-rich motifs such as those of TIA1, ELAVL1, and HNRNPC, showed stronger enrichment 10–20 bp upstream of CS micro-exons than for longer exons (Supplemental Fig. 11). However, the largest difference between CS micro-exons and longer CS exons was found for the binding motif of U2AF2 (Fig. 4D). As many as 96% of all CS micro-exons possess a U2AF2-like motif 10 to 20 bp upstream of their splice sites (Methods). U2AF2 is known to bind to the polypyrimidine tract and is necessary for spliceosome maturation and pre-mRNA splicing (Abovich and Rosbash 1997; Kent et al. 2005). The precision by which U2AF2 binds immediately upstream of CS micro-exons may therefore further enhance accurate splicing.

### The intronic flanks of AS micro-exons harbor conserved splicing signals

**Figure 5.**
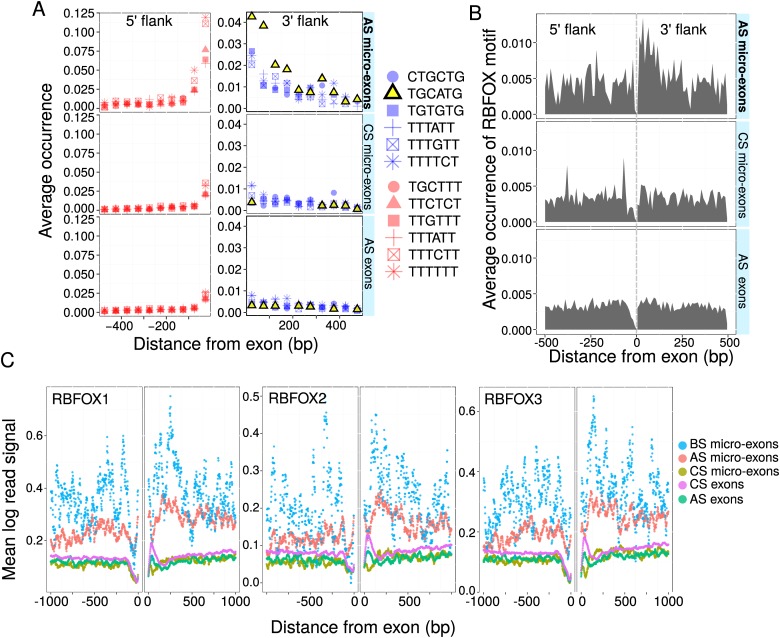
Conserved motifs and splice-factor binding sites. (*A*) The 6-mer corresponding to the RBFOX protein family motif (TGCATG) is highly overrepresented in introns downstream from AS micro-exons compared to other classes of exons. Average occurrence of conserved 6-mers in the intronic flanks of exons. Conserved 6-mers were computed according to an entropy threshold based on multiple sequence alignments including human, *Rhesus macaque*, mouse, cattle, and dog sequences. Of all conserved 6-mers, pyrimidine-rich 6-mers were found to be enriched in the intronic sequences immediately upstream of exons belonging to all classes, with a higher enrichment upstream of alternatively spliced (AS) micro-exons. (*B*) In human, the RBFOX-binding motif (TGCATG) is overrepresented in the intronic sequences downstream from AS micro-exons. (*C*) Analysis of RBFOX protein CLIP-seq data sets in mouse brain shows that AS micro-exons and AS micro-exons that are brain specific (BS micro-exons) each possess a higher number of RBFOX binding events in their intronic flanks than other types of exons.

We observed earlier that intronic flanks of symmetric AS micro-exons were highly conserved and proposed that they may harbor regulatory signals. We therefore conjectured that, unlike CS micro-exons that show elevated density of splicing enhancers within their exonic sequence, it is within intronic flanks that AS micro-exons harbor splicing enhancers that facilitate their recognition by the splicing machinery. To detect such signals of splicing, we aligned human exons and their intronic flanking sequences to the genomes of four other mammalian species: *Rhesus macaque*, cattle, dog, and mouse (Methods). Splicing motifs are generally 4–10 nt in length (Fairbrother et al. 2002). We therefore searched for conserved 6-nt motifs that are overrepresented near AS micro-exons. To this end, we computed the entropy for each gapless 6-nt sliding window (Methods) and searched for 6-mers with entropy in the lowest 10-percentile of the empirical distribution (entropy *<* 1.0). In the 5′ intronic flank of AS micro-exons, CS micro-exons, and all AS exons, several pyrimidine-rich motifs were found to be overrepresented (Fig. 5A). Interestingly, only one motif, TGCATG, was found to be highly overrepresented in the 3′ intronic flanks of AS micro-exons. Notably, this overrepresentation is absent from the intronic flanks of CS micro-exons and longer exons and is therefore unique to the 3′ intronic flanks of AS micro-exons.

TGCATG (or UGCAUG) is a well-characterized motif that is bound by RBFOX splicing factors (Zhang et al. 2008; Lovci et al. 2013). We therefore sought to determine the spatial distribution of the motif in the human genome. We observed up to twofold enrichments in the number of RBFOX binding motifs in the immediate 3′ flanks of AS micro-exons compared to other classes of exons (Fig. 5B). This supports a role of RBFOX proteins in the splicing of AS micro-exons.

### The inclusion of AS micro-exons is likely regulated by RBFOX proteins and PTBP1

Next we hypothesized that AS micro-exons are regulated by RBFOX binding events in their intronic flanks, which possibly act by enhancing splicing. Indeed, we found an unexpectedly higher density of experimentally determined RBFOX binding events near AS micro-exons, most prominently in their 3′ flanks. We also found that this density is even higher for the 145 AS micro-exons that are brain specific. To do this, we first obtained CLIP-seq replicate data sets for all three RBFOX family members (RBFOX1, RBFOX2, RBFOX3) from mouse brains (Weyn-Vanhentenryck et al. 2014). We then projected all exons from the different exon classes to the mouse genome and computed read densities near exons for each class (Fig. 5C; Methods). All three RBFOX family members exhibit the same binding patterns: a higher density of reads in the intronic flanks of 145 brain-specific AS micro-exons (Methods) and, to a lesser extent, in the intronic flanks of all AS micro-exons compared to other classes of exons.

The higher density of RBFOX binding events in the 3′ intronic flanks compared to the 5′ intronic flanks of AS micro-exons encouraged us to investigate the putative role of RBFOX proteins in enhancing micro-exon splicing. This is because binding of RBFOX proteins downstream from alternatively spliced exons is known to enhance exon inclusion (Witten and Ule 2011; Han et al. 2013; Weyn-Vanhentenryck et al. 2014). Furthermore, we noted the presence of several micro-exons whose inclusion appears to be specific to brain, heart, and muscle transcripts (Fig. 2). RBFOX proteins are known to be exclusively expressed in neurons, heart, and muscle (Weyn-Vanhentenryck et al. 2014). Although these observations support a critical role of RBFOX proteins in the regulation of micro-exons, it is likely that other splicing factors regulate the splicing of micro-exons in a cell-type-specific manner. For example, the splicing factors ESPR1 and ESPR2 play important roles in regulating epithelial cell-type-specific splicing (Warzecha et al. 2009). However, when we computed the ESRP2 motif occurrence in intronic sequence adjacent to brain-expressed AS micro-exons (data not shown), we observed no clear enrichment in contrast to what we observed for RBFOX or PTBP1 motifs. This motivated us to focus on RBFOX and PTBP1.

**Figure 6.**
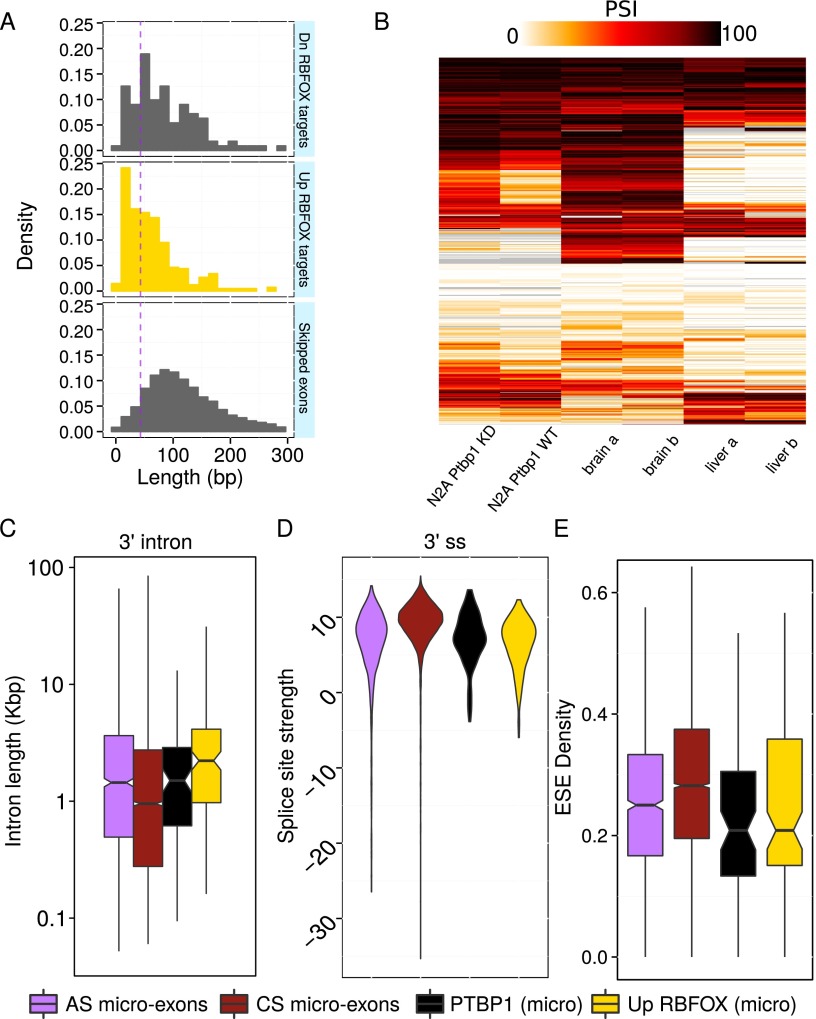
RBFOX proteins and PTBP1 regulate micro-exon usage. (*A*) Exons whose inclusions are enhanced by RBFOX (yellow) tend to be shorter than those that are repressed by RBFOX and alternatively spliced exons in mouse. Data from Weyn-Vanhentenryck et al. (2014). RBFOX targets also tend to be shorter than AS exons. (*B*) *Ptbp1* knockdown (KD) in N2A neuroblastoma cell lines leads to a widespread increase in micro-exon inclusion compared to wild type (WT). Heatmap of 707 micro-exons alternatively spliced in mouse. Data from Han et al. (2014). Compared to other micro-exons, micro-exons up-regulated by RBFOX proteins tend to possess (*C*) longer flanking introns than other micro-exons, (*D*) weaker splice sites, and (*E*) lower densities of exonic splicing enhancers (ESE) (see also Supplemental Fig. 13 for 5′ intron and splice-site strength distributions). Micro-exons whose inclusions are repressed by PTBP1 possess (*D*) weaker splice sites and (*E*) lower densities of exonic splicing enhancers.

To further understand the connection between RBFOX proteins and micro-exon splicing, we analyzed 521 AS exons that were either predicted to be enhanced (410 micro-exons) or repressed (111 micro-exons) in the brain by the proximal binding of RBFOX (Weyn-Vanhentenryck et al. 2014). We found that the size distribution of AS exons enhanced by RBFOX binding was markedly different from that of AS exons repressed by RBFOX binding (Fig. 6A). In particular, AS exons enhanced by RBFOX binding were much shorter and were more likely to be < 51 nt in length, i.e., micro-exons (Fig. 6A). Indeed, whereas only 8.6% of skipped exons were predicted to be micro-exons (Methods), 22.5% and 41.7% of repressed and enhanced targets of RBFOX proteins were micro-exons. This supports the notion that splicing factors may simultaneously aid the regulation and the recognition of AS micro-exons by binding to splicing motifs located in their intronic flanks.

Owing to several pyrimidine-rich motifs conserved upstream of AS micro-exons, we also investigated the possibility that micro-exons were regulated by the binding of splice factors to their polypyrimidine tracts. Previously, Xue et al. (2013) showed that a 12-nt micro-exon in *KDM1A* was repressed by the polypyrimidine tract binding protein 1 (PTBP1): Knocking down *PTBP1* resulted in a higher inclusion of the 12-nt micro-exon. Similarly to the RBFOX CLIP-seq study, our analysis of this PTBP1 CLIP-seq data from HeLa cells revealed a higher density of binding near AS micro-exons (Supplemental Fig. 12). Furthermore, the signal enrichment immediately upstream of AS micro-exons is consistent with binding to the polypyrimidine tract. To further investigate the regulation of micro-exons by PTBP1, we obtained a RNA-seq data set consisting of wild-type and *Ptbp1* knockdown mouse N2A neuroblastoma cell line samples (Han et al. 2014). After mapping the reads onto mouse micro-exons using ATMap, we first observed that N2A micro-exons share similar inclusion patterns to those of mouse brain (Fig. 6B). We then asked whether there were micro-exons that show differential inclusion rates between wild-type and *Ptbp1* knockdown, and if so whether there was an excess of micro-exons that appear to be repressed by PTBP1. We found that 141 micro-exons showed differential inclusion (|ΔPSI| > 15) of which a vast majority (129 micro-exons, ∼92%) increased in inclusion following *Ptbp1* knockdown. PTBP1, unlike RBFOX proteins, may therefore regulate micro-exons by repressing their inclusion either directly by binding to the polypyrimidine tract or indirectly by regulating other splice factors.

We next wished to study the genomic features and inclusion of micro-exons regulated by RBFOX proteins and PTBP1. To this end, we projected the 171 micro-exons enhanced by RBFOX and the 129 micro-exons repressed by PTBP1 from the mouse genome to the human genome that resulted in 159 and 113 human RBFOX- and PTBP1-regulated micro-exons, respectively. Interestingly, if we assume that all 1480 AS micro-exons may be targeted by RBFOX proteins and by PTBP1, the number of micro-exons predicted to be both enhanced by RBFOX proteins and repressed by PTBP1 (25) is significantly larger than expected by chance (*P*-value *<* 6.7 × 10^−5^; hypergeometric test). This suggests that PTBP1 may act coordinately with RBFOX proteins and perhaps other splicing factors to accurately regulate micro-exon inclusion. When we compared the intron length, splice-site strength, and exonic splicing enhancer density of RBFOX- and PTBP1-regulated micro-exons, we found a general trend of “weakened” splicing features compared to other micro-exons (Fig. 6C,D,E; Supplemental Fig. 13). For example, intronic flanks of micro-exons regulated by RBFOX proteins tend to be longer than those of AS micro-exons (3′ intron median length 2.2 kb vs 1.4 kb; *P*-value *<* 0.001) and possibly those of AS exons (2.2 kb vs. 1.8 kb; *P*-value = 0.052). The density of exonic splicing enhancers was also significantly lower for PTBP1-regulated micro-exons (median 0.208 vs. 0.250–0.286; *P*-value *<* 0.01; Mann-Whitney *U*-test). The binding of factors to these intronic splicing enhancers alone may therefore compensate for the difficulty in processing very short exons.

### Alternative inclusion of micro-exons can alter protein–protein interactions

**Figure 7.**
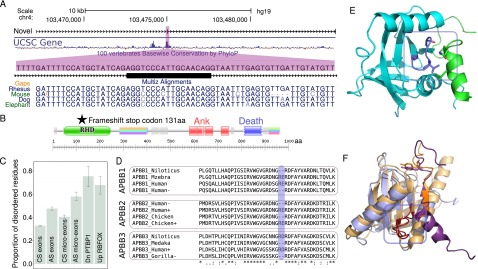
Alternatively spliced micro-exons and molecular functions. (*A*) UCSC Genome Browser view of a novel 17-nt micro-exon in *NFKB1*. This micro-exon is located within a well-conserved region and possesses splice sites that are conserved in mammals. (*B*) Inclusion of this micro-exon induces a frameshift and a premature stop codon in the 5′ region of the *NFKB1* pre-mRNA, which possibly triggers nonsense-mediated decay. (*C*) Alternatively spliced micro-exons tend to fall within intrinsically disordered regions more than other classes of exons (bars represent 95% confidence intervals). (*D*) Translated cDNA sequences from proteins belonging to the APBB protein family. A 6-nt micro-exon exists at a paralogous position in all three *APBB* genes. Furthermore, all three proteins possess two isoforms, one with the micro-exon included and one without, in both mammals (human, gorilla), birds (chicken), and fish (Niloticus: *Oreochromis niloticus*, Mzebra: *Metriaclima zebra*). (*E*) 3D structure of the phosphotyrosine binding domain of APBB2 (cyan) in a complex with the interacting amyloid-beta protein (green). The close proximity of the cytoplasmic tail of the amyloid-beta protein to the loop containing the two amino acid residues suggests that they interact (blue box). (*F*) Superposition of the phosphotyrosine binding domain of APBB2 (light blue) and of the LDLRAP1-PTB domain (orange). After TNS4 was mapped onto the structure of the LDLRAP1-PTB domain, we found that both *APBB2* and *TNS4* possess a micro-exon that encodes residues mapping to homologous loops (red). The inclusion or exclusion of these amino acid residues are therefore expected to both alter the interactions of APBB2 and TNS4 with amyloid-beta protein and beta integrin, respectively.

Alternatively spliced exons contribute greatly to protein diversity (Black 2000; Romero et al. 2006). We therefore sought to characterize the impact of micro-exon inclusion or exclusion on protein structure. We first quantified the proportion of micro-exons that have coding potential, i.e., they do not introduce in-frame stop codons (Methods). Overall, we found that at least 96.0% (5247 out of 5463) and 78.7% (1244 out of 1581) of CS and AS micro-exons, respectively, have the potential to encode for amino acids. Interestingly, up to 22% of AS micro-exons thus may introduce in-frame stop codons when spliced in. For example, we identified a novel 17-nt micro-exon in *NFKB1* that lies in a conserved genomic region and is predicted to generate a frameshift when included in the canonical *NFKB1* transcript (Fig. 7A,B). Regulated inclusion of AS micro-exons, in particular those that are not symmetric, may therefore play a role in post-transcriptional regulation, especially in nonsense-mediated decay. This is consistent with the elevated sequence conservation in the intronic flanks of nonsymmetric AS micro-exons compared to other classes of exons (Supplemental Fig. 8).

AS micro-exons that had coding potential (∼80% of all AS micro-exons) encoded peptides that were preferentially situated within intrinsically disordered regions of proteins (DISOPRED: 54.4–61.9, 95% confidence interval) compared to CS micro-exons (46.4%–49.0%) and longer exons (32.5%–41.2%; Methods) (Fig. 7C). Remarkably, 75.5% (65.5%–83.8%) and 67.8% (60.1%–75.3%) of residues from micro-exons that are targeted by PTBP1 and RBFOX proteins lie within disordered regions. Since alternative splicing of disordered regions is known to rewire interaction networks in a tissue-specific manner (Romero et al. 2006; Buljan et al. 2012), an important role of AS micro-exons, and in particular those that are regulated by PTBP1 and RBFOX proteins, might be to alter protein–protein interactions.

To explore whether AS micro-exons could alter protein–protein interactions, we searched for AS micro-exons that encode residues from domains known to interact with other proteins. Among genes with conserved AS micro-exons with coding potential, we identified a paralogous gene family consisting of three amyloid binding proteins: APBB1, APBB2, and APBB3. All three APBB (amyloid-beta [A4] precursor protein-binding, family B) genes possess 6-nt micro-exons encoding two amino acids located within the first of two phosphotyrosine-binding (PTB) domains. Searching cDNA databases revealed that the micro-exons within APBB are also present and alternatively spliced in fishes, in chicken, and in diverse mammals (Fig. 7D). These micro-exons are located at paralogous positions within APBB genes, indicating that these micro-exons have survived > 400 My of evolution since these genes duplicated in early vertebrate evolution (Fig. 7D).

To investigate the functional impact of these micro-exons on protein structure, we mapped the two amino acid residues encoded by the micro-exon onto the known 3D structure of APBB2 (Fig. 7E). This showed that the two residues are located in a beta-turn loop of APBB2, which we predict to interact with the cytoplasmic tail of amyloid-beta protein from their close proximity in the complex (Fig. 7C). Interestingly, all four protein members from the tensin (TNS) family also possess micro-exons (Supplemental Fig. 14) which encode residues within their phosphotyrosine-binding domains. By mapping the TNS4 sequence onto a homology model (Methods), we found that, despite their sequence dissimilarity, tensin micro-exons map to the same beta-turn as the amino acids encoded by micro-exons of the APBB protein family (Fig. 7F). This points toward a mechanism that is conserved across divergent homologs that controls protein–protein interactions through the alternative inclusion of micro-exons.

## Discussion

The splicing process requires a large number of steps, each of which depends on multiple proteins and genome-encoded signals (Graveley 2000). Previous studies reported compensatory relationships between diverse genomic features that are thought to facilitate splicing (Graveley 2000; Dewey et al. 2006). For example, enhancer-dependent splicing events can be relieved of their enhancer requirement by increasing the ability of the polypyrimidine tract to recruit binding of U2AF (Tian and Maniatis 1994). Conversely, artificially weakening of the polypyrimidine tract, and thus U2AF binding affinity, can make the efficient splicing of an exon more dependent on splicing enhancers (Tian and Maniatis 1992; Graveley and Maniatis 1998). Furthermore, splice-site strengths and exonic splicing enhancer densities were found to be positively correlated with intron sizes in several organisms (Fields 1990; Weir and Rice 2004; Dewey et al. 2006), suggesting that exons flanked by long introns require compensatory signals for their efficient splicing. Efficient splicing thus appears to depend on several genomic features, and a strong genomic feature also appears to be able to compensate for another weaker one to maintain efficient splicing.

It has become increasingly clear over recent years that splicing is tightly linked to human diseases (Padgett 2012; Singh and Cooper 2012), and it has even been suggested that the majority of mutations that cause disease do so by disrupting splicing (Lopez-Bigas et al. 2005). Here, we presented data suggesting that 80% of brain-expressed micro-exons are well conserved in vertebrates and are thus expected to be functional. Therefore, understanding the splicing mechanisms that facilitate their processing will not only allow us to better understand splicing regulatory mechanisms, but it may also reveal novel disease etiologies. This is most relevant for very short exons that may be particularly susceptible to dysregulation in splicing.

Based on previous studies (Black 1991; Dominski and Kole 1991), we expected micro-exons to be difficult to process owing to their short sizes. To our surprise, we found that a large majority of micro-exons were constitutively spliced, and therefore efficiently processed. Upon investigation, we discovered that constitutively spliced micro-exons tend to (1) be flanked by shorter flanking introns, (2) possess stronger splice sites, and (3) harbor a higher density of exonic splicing enhancers. More notably, 96% of CS micro-exons possessed a motif that is associated with U2AF binding within 10–20 nt upstream (compared to only 61% for longer CS exons). The short sizes of most micro-exons are therefore compensated by multiple genomic features that enhance their recognition by the splicing machinery.

Despite the presence of compensatory features for a large class of 6469 CS micro-exons, we observed that the 1480 AS micro-exons generally did not possess such splicing-enhancing features. Instead, they were characterized by highly conserved intronic flanks that harbor conserved intronic splicing enhancers. We identified RNA-binding proteins, RBFOX proteins, and PTBP1, which act as preferential regulators of micro-exons. Indeed, we confirmed that RBFOX proteins and PTBP1 bind with higher affinity near AS micro-exons compared to other exons. In addition to regulating inclusion ratios of micro-exons, RBFOX proteins appear to also enhance the splicing efficiency of micro-exons. We observed that micro-exons that are predicted to be targets of RBFOX proteins or PTBP1 possess weaker splicing features than other AS or CS micro-exons. This observation, coupled with the finding that most micro-exons appear to require compensatory genomic features to maintain splicing, suggests that the splicing-enhancing activity of RBFOX proteins alone is able to guarantee efficient splicing. Our results that micro-exons targeted by PTBP1 also possessed weak splicing signals were somewhat surprising. This is because PTBP1 generally functions as a splicing repressor, possibly through competition with U2AF2 to bind the polypyrimidine tracts of micro-exons. However, we found that 25 of the 113 PTBP1 targets were also predicted to be enhanced by RBFOX proteins. Given that the set of exons predicted to be targets of RBFOX proteins is likely a subset of all RBFOX targets (Weyn-Vanhentenryck et al. 2014), it is possible that other PTBP1-regulated micro-exons are also targeted by RBFOX proteins or other RNA-binding proteins that enhance splicing. Overall, the efficient splicing of AS micro-exons appears to depend on intronic splicing enhancers that promote binding of splice factors, rather than on general splicing signals that we observed for CS micro-exons.

Each micro-exon only encodes a small number of amino acid residues. We thus sought to better understand whether and how they might affect the structure and function of a protein. We speculated that alternatively spliced micro-exons, much like longer AS exons, could generate multiple protein isoforms with distinct functions. This hypothesis is supported by the strong bias for micro-exons to be exon-phase symmetric. We further found that amino acid residues encoded by AS micro-exons, and in particular those that are targeted by RBFOX proteins and PTBP1, preferentially lie within intrinsically disordered regions. This suggests that many AS micro-exons could alter protein–protein interactions. (Romero et al. 2006; Vavouri et al. 2009; Babu et al. 2011). Our analyses are the first, to our knowledge, to identify several conserved AS micro-exons that can alter protein–protein interactions in a switch-like manner. For example, all three members of the *APBB* gene family and all four members of the tensin (*TNS*) gene family possess alternatively spliced micro-exons mapping to their beta-turn loops. The alternative inclusion or exclusion of these micro-exons lengthens or shortens the beta-turn loop, and is therefore expected to alter protein–protein interactions: APBB with amyloid-beta protein and TNS with beta integrin. In light of these findings, we propose that alternate use of micro-exons often alters local binding-site specificities, whereas the inclusion of longer AS exons may more substantially alter global protein structure. Although we show that several AS micro-exons are likely to regulate protein–protein interactions, we believe it is premature to conclude that this is their canonical function. In fact, it is likely that micro-exons also function through other mechanisms. For example, a 13-nt micro-exon that is alternatively included near the 3′ end of *Arl6* transcripts was found to be required for vision in mammals (Pretorius et al. 2010, 2011). The inclusion of this micro-exon induces a frameshift that precludes an ARL6 protein isoform with a completely different C-terminal sequence.

The finding that AS micro-exons, especially those that are regulated by RBFOX proteins, can alter protein–protein interaction is noteworthy in the context of disease. RBFOX proteins are well known to play crucial roles in both brain development and function (Gehman et al. 2011, 2012; Weyn-Vanhentenryck et al. 2014). Additionally, aberrant splicing induced by RBFOX dysregulation is associated with a variety of brain-related disorders including autism, mental retardation, and epilepsy (Gehman et al. 2012; Weyn-Vanhentenryck et al. 2014). Therefore, our finding that an unexpectedly large proportion of exons targeted by RBFOX proteins are very short, i.e., micro-exons, hints at an involvement of micro-exon dysregulation in brain-related diseases. Furthermore, while longer exons may be less dependent on the enhancing effects of RBFOX proteins, accurate splicing of AS micro-exons may depend more heavily on RBFOX activity because they tend to lack compensatory features that other short exons appear to require for efficient processing. Splicing of AS micro-exons regulated by RBFOX proteins may therefore be among the most susceptible to dysregulation.

In summary, this study has revealed an unexpectedly large number of functional micro-exons and has shed light on how they may be accurately spliced. Our findings should encourage further studies into the links between RNA-binding proteins (RBFOX proteins and PTBP1) and micro-exon splicing in the context of both splicing regulation and brain-related disorders.

## Methods

### Data set retrieval

This study uses transcriptome data in the form of 76-bp paired-end RNA-seq reads from 345 postmortem prefrontal cortices, 74 muscle samples, 47 nerve samples from human, and up to 10 samples for other tissues within the GTEx (Lonsdale et al. 2013), 76-bp paired-end reads from 25 postmortem human brain across development and aging (Mazin et al. 2013) and from diverse human organs from the Illumina Human BodyMap Project (European Nucleotide Archive [ENA; http://www.ebi.ac.uk/ena/]; accession number ERA022994); mouse transcriptome data were obtained from Merkin et al. (2012). A list of all RNA-seq libraries used can be found in Supplemental File 3 with their accession numbers. RBFOX CLIP-seq data were obtained from mouse brains (Weyn-Vanhentenryck et al. 2014).

### Discovery and mapping of splicing events

To identify novel micro-exons from RNA-seq data, 307 brain samples, 74 muscle, and 47 nerve samples from GTEx were mapped onto Ensembl (release 70) (Flicek et al. 2014) cDNA transcripts using Stampy (Lunter and Goodson 2011), allowing for multiple mapping locations (options–xa-max = 5 -t4 -v3). In the filtering step, only reads mapping with an insertion of size 3 to 51 nt that are flanked by at least 6 nt matches on both sides were retained. Subsequently, insertions overlapping exon–exon boundaries were retrieved, and those supported by fewer than 10 reads were discarded. Introns separating exon–exon boundaries were then scanned for the inserted sequences. Sequences flanked by the canonical splice sites were then considered to be putative micro-exons. In the case of ambiguous mapping, a location at random was chosen to represent the putative micro-exon. Of 7575 inserted sequences, only four inserted sequences 6 nt or longer were found to map to ambiguous sites (< 0.1%), but 297 sequences of 3 nt in length were found to be ambiguous (21.7%). We further required micro-exons to be expressed in at least 15% of all samples coming from each tissue (i.e., each micro-exon must have at least 5% PSI in 45 of 335 brain samples) in order to be considered novel. To allow comparison between novel predicted micro-exons and previously annotated micro-exons, we also required previously annotated micro-exons to have the same expression breadth as novel micro-exons.

An in-house pipeline was developed in order to quantify micro-exon usage. A transcriptome augmented by alternatively spliced micro-exons was created as follows: (1) All micro-exons were identified from an annotation file, (2) for each transcript, we constructed a version with the micro-exon(s) included and another without, and (3) in cases where there were multiple micro-exons in the same gene, we constructed transcripts representing all possible combinations of micro-exon inclusion/exclusion for those that are 100 nt within one another. Step (3) was important for quantifying alternatively spliced micro-exons located in tandem (several collagen genes harbor multiple micro-exons). RNA-seq data sets were then mapped to this augmented transcriptome using BWA (single-end; options samse -n 100), allowing at most two mismatches per read. The quantities *R*_*L*_ and *R*_*R*_, representing the number of reads supporting the left and right junction, respectively, were then computed by counting the number of reads that span each junction. According to these quantities, the number of reads supporting each micro-exon, *R_tot_*, was then computed using the following equation:
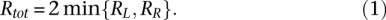
In this case, taking the minimum of these two quantities avoided cases in which an alternative 5′ or 3′ splice site biases the estimated micro-exon usage. Using *R*_*tot*_, the percent spliced-in statistic was computed for each micro-exon: *R_tot_/(R_tot_+R_skipped_)*, where *R_skipped_* represented the number of reads supporting an exon skipping event.

To compare our in-house pipeline to STAR (Dobin et al. 2013), TopHat2 (Kim et al. 2013), and OLego (Wu et al. 2013), paired-end reads from a 76-nt postmortem human brain sample (SRR112675) (Mazin et al. 2013) were mapped using STAR with standard options and maximum two mismatches (-M 2), TopHat2 with both standard options and micro-exon-search, and OLego with standard options. The numbers of reads supporting micro-exons were then computed according to the equation above.

### Identifying brain-specific micro-exons

Brain-specific micro-exons were defined to be those that have a median PSI of at least 25 in GTEx brain samples, and an 80 percentile of at most 10 PSI in other samples, excluding samples from pituitary gland (due to their relatedness to brain). A total of 145 brain-specific micro-exons were identified using these thresholds, but with 120–200 using different cut-offs.

### Finding a set of constitutive and alternatively spliced exons

To identify a set of control exons to allow comparison with micro-exons analyzed in this study, 100 samples from the GTEx brain were chosen randomly and analyzed. Spliced reads were recovered from each of the samples and the PSI of each internal exon was computed in the same way as for internal micro-exons. Exons with median PSI at least 10% and at most 90% were classified as alternatively spliced, while exons with PSI higher than 90% were classified as constitutively spliced.

We explored several PSI criteria for defining AS: 5%, 10%, 15% as the lower bound and 85%, 90%, 95% as the upper bound. Our first observation was that the qualitative interpretation of our results was independent of the criteria used. Our second observation, however, was that exons (both long and short) with median PSI 5%–10% in our samples were less conserved (with respect to Vertebrate phastCons scores, data not shown) than exons with higher median PSI. This motivated us to use 10% PSI as a lower bound.

### Conservation of micro-exons

To characterize the sequence conservation of micro-exons and their intronic flanks, micro-exons were centered within 300-nt windows and vertebrate phastCons scores (from UCSC Genome Browser, version hg19) were retrieved for each position. phastCons confidence intervals for different classes of exons were then computed by bootstrapping as such: (1) Let *S* denote a set containing vectors of size 300, each representing the phastCons score of one exon at each of the 300-nt positions, (2) draw randomly with replacement |*S*| vectors from this set, (3) compute the average conservation profile *S*_*i*_, (4) repeat this process 1000 times, obtaining {*S*_*i*_: 1 ≤ *i* ≤ 1000}. The lower and upper values of the confidence interval for each position 1 ≤ *k* ≤ 300 correspond to the 5- and 95-quantiles of {*S*_*i*_(*k*): 1 ≤ *i* ≤ 1000}, respectively.

To compute the percent identity between human brain-expressed exons and orthologs in dog, mouse, platypus, and chicken, the internal human exons and their intronic flanks were aligned to corresponding orthologous sequences. The percent identity between human and the other species was then computed for 100 nt of intronic sequences flanking the 5′ and 3′ ends of each exon. Additionally, the percent identity of 12 nt from the exon (6 nt from both the 5′ end and 3′ end) was also computed to remove biases from differing exon lengths.

### Identification of conserved and overrepresented *k*-mers

In order to identify conserved *k*-mers, all regions consisting of human internal exons plus 500 nt intronic flanking sequences (250 nt from each flanking intron) were collected. These regions were projected onto the genomes of rhesus monkey, cattle, mouse, and dog using liftOver, and were then aligned using MUSCLE (Edgar 2004). Subsequently, the resulting multiple sequence alignments were divided into three regions: 5′ intronic sequences, exonic sequences, and 3′ intronic sequences. A sliding window of size 6 nt was then used to scan for conserved regions, which correspond to gapless 6-nt alignments with entropy scores > 1.0. This entropy threshold corresponds to the 10% most conserved 6-mers. Different entropy thresholds were found to yield very similar results. Here, entropy was computed as −∑ _*i*=1_^6^*f*_*a,i*_ log *f*_*a,i*_, where *f*_*a,i*_ is the relative frequency of base *a* at position *i* of the multiple sequence alignment.

### CLIP-seq data set analysis

To determine the enrichment of RBFOX protein-binding events near different classes of exons, raw CLIP-seq reads were obtained from (SRP039559) and mapped using BWA (Li and Durbin 2009) to the mouse genome (mm10). Replicates were then merged and the average number of reads mapped was computed for each position flanking different classes of exons. For PTBP1, bed coverage of PTBP1 CLIP-seq was obtained from GEO (GSM1048186) (Xue et al. 2013).

### RNA-binding compendium analysis

To identify motifs that were enriched upstream of CS micro-exons, the binding affinity of 131 human RNA-binding proteins (RBP) were computed using a sliding window of 7 bp, and were based on position weight matrices (7 nt) derived from experimental data (Ray et al. 2013). We tested several thresholds over which we defined 7-nt sequences to allow RBP binding, and used a threshold of 7 × log(0.6) as a lower bound. T-rich motifs were then scanned through to identify differences in affinity between the upstream (5′) flanking regions of different classes of exons. Note that changing the threshold does not change the qualitative differences between CS micro-exons and other classes of exons that we observed.

### Micro-exons, protein domains, and tertiary structures

All 8891 protein sequences containing a brain-expressed internal exon studied here were collected and disordered regions were predicted using DISOPRED (Jones and Ward 2003). The disorder of 8308 proteins were successfully predicted at a residue-level resolution, and were used to compute the proportion of disordered and coiled residues in the different classes of exons.

To identify micro-exons overlapping protein domains, HMMSCAN (Eddy 2010) was used with Pfam-A HMM database. The protein structure of APBB2 was retrieved from PDB with ID 2ROZ. To identify a structure homologous to the TNS4 phosphotyrosine-binding (PTB) domain, the HHpred server for structure prediction (Soding et al. 2005) was used with the TNS4 PTB domain sequence as input (SwissProt ID: TENS4_HUMAN, ACC: Q8IZW8, amino acids 581–714). The crystal structure of LDLRAP1 (low density lipoprotein receptor adaptor protein 1) PTB domain in complex with the LDL receptor (LDLR) tail (PDB ID: 3SO6) was then considered to be the most appropriate proxy for the TNS4 PTB domain structure prediction and analysis of its putative interaction surface (Dvir et al. 2012). Micro-exons within each protein were then mapped onto their corresponding structure using PyMol (http://www.pymol.org/). Structural superposition of PTB domains was performed using Dali (Holm and Sander 1995).

## Supplementary Material

Supplemental Material
