# Supplementary material for: RBFOX and PTBP1 proteins regulate the alternative splicing of micro-exons in human brain transcripts
Source: Genome Res. 2015 Jan;25(1):1–13. doi: 10.1101/gr.181990.114 (PMC4317164; doi:10.1101/gr.181990.114)
Supplement: Supplemental Material [file supp_25_1_1__index.html]

RBFOX and PTBP1 proteins regulate the alternative splicing of micro-exons in human brain transcripts — Supplemental Material 

# RBFOX and PTBP1 proteins regulate the alternative splicing of micro-exons in human brain transcripts

## Supplemental Material

**Files in this Data Supplement:**

- Supp Figures.pdf
- Supp File 1.txt
- Supp File 2.txt
- Supp File 3.txt
- Supp Legends.docx
