# Supplementary material for: RBFOX and PTBP1 proteins regulate the alternative splicing of micro-exons in human brain transcripts
Source: Genome Res. 2015 Jan;25(1):1–13. doi: 10.1101/gr.181990.114 (PMC4317164; doi:10.1101/gr.181990.114)
Supplement: Supplemental Material [file supp_gr.181990.114_Supplemental_Legends.docx]

Supplementary Figure 1: Vertebrate conservation of micro-exons and exons in general.

(A) Vertebrate conservation profiles of 6, 9 and 12nt Ensembl-annotated versus novel predicted micro-exons. (B) Vertebrate conservation heatmap of 1,500 randomly sampled constitutively spliced (CS) and alternatively spliced (AS) exons. Each exon was centred within a 300nt window.

Supplementary Figure 2: 3nt novel micro-exon in *TLN1*.

Example of a conserved 3nt micro-exons in *TLN1*. The splice sites are conserved in mammals.

Supplementary Figure 3: Mapping RNA-seq reads onto micro-exons

The short pair-end reads (76bp) from a human brain sample (SRR112675) were mapped onto the Ensembl (release 70) annotated human genome with ATMap, STAR, TopHat2, TopHat2 with “micro-exon-search” activated (TOPHATm), and OLego. For all exons of sizes 3 to 51bp, we computed the pairwise differences in inclusion predictions and binned them by exon sizes. Only symmetric exons (i.e. exons of sizes that are a multiple of 3) are displayed as they consist of a larger number of cases. Note: The difference between OLego and ATMap’s mapped read counts onto micro-exons shorter than 10nt is largely caused by a default minimal size (9nt) for the micro-exon search step in OLego.

Supplementary Figure 4: Constitutively and alternatively spliced micro-exons in the human brain.

Histogram of constitutively (CS) and alternatively (AS) spliced micro-exons depending on size according to GTEx brain samples. While the number of CS micro-exons sharply decreases as exon size decreases, the number of AS micro-exons remains relatively constant, particularly those whose size is an integer multiple of three.

Supplementary Figure 5: Inclusion of micro-exons in all analysed tissues.

PSI of 22–51nt micro-exons (A) and 6nt–21nt micro-exons (B) in all analysed tissues, including GTEx samples, Illumina Human BodyMap Project (European Nucleotide Archive [ENA; <http://www.ebi.ac.uk/ena/>]; study accession number ERA022994) samples, and samples from developing and ageing postmortem human brains ([Mazin et al.](#Xpmid23340839), [2013](#Xpmid23340839)). (C) Inclusion ratios of 145 AS micro-exons predicted to be brain-specific. In gray: micro-exons for which PSI could not be computed because of insufficient number of reads (≤5) spanning splice junctions.

Supplementary Figure 6: Pearson correlation of micro-exon usage levels

Pearson correlation of micro-exon PSI among human brain samples, between human brain and other human samples, between human and mouse brain samples and between mouse brain and other mouse samples.

Supplementary Figure 7: Sequence percent identity of exons and their flanking introns.

Percent identity of the pairwise aligned intronic sequences and exonic sequences of different classes of exons between human, and rhesus monkey, mouse, platypus, and chicken. The nucleotide percent identity follows an increasing trend with decreasing exon sizes for both AS and CS exons in intronic flanking regions. However, only AS micro-exon exonic sequences show a difference in percent identity compared to other classes of exons.

Supplementary Figure 8: Sequence conservation in the vicinity of symmetric (sym) versus non-symmetric (ns) micro-exons that are alternatively (AS) or constitutively (CS) spliced.

Supplementary Figure 9: (A) Exonic enhancer density appears to be constant for micro-exons of difference sizes. However, (B) there appears to be a small increase in splice site strengths (MaxEntScan score) as exon size decreases for CS micro-exons. We must note here that the robustness of this observation is questionable owing to the very limited number of very short CS micro-exons.

Supplementary Figure 10: Nucleotide content in the flanks of exons.

Supplementary Figure 11: Enrichment of T-rich RNA-binding motifs.

Thymine-rich motifs are more strongly over-represented upstream (5’) of CS micro-exons compared to other classes of exons.

Supplementary Figure 12: PTBP1 CLIP-seq binding

Increased binding of PTBP1 near AS micro-exons in HeLa cells. In contrast to RBFOX protein CLIP-seq, the binding density appears to be higher immediately upstream of AS micro-exons. Data from ([Xue et al.](#Xpmid23313552), [2013](#Xpmid23313552)).

Supplementary Figure 13:

Compared to alternatively spliced (AS) micro-exons, micro-exons upregulated by RBFOX proteins tend to possess (A) longer flanking introns and (B) weaker 3’ splice sites. Micro-exons repressed by PTBP1 tend to have weaker splice sites (B) compared to other micro-exons.

Supplementary Figure 14: Tensin alignments

Regional alignment of human proteins belonging to the tensin family. Residues encoded by micro-exons are highlighted in red. Gaps are indicated by “-” symbols.
